# Real Time Metagenomics: Using *k*-mers to annotate metagenomes

**DOI:** 10.1093/bioinformatics/bts599

**Published:** 2012-10-09

**Authors:** Robert A. Edwards, Robert Olson, Terry Disz, Gordon D. Pusch, Veronika Vonstein, Rick Stevens, Ross Overbeek

**Affiliations:** ^1^Mathematics and Computer Science Division, Argonne National Laboratory, Argonne, IL 60439, USA, ^2^Department of Biology, ^3^Department of Computer Science, San Diego State University, San Diego, CA 92182, USA, ^4^Computation Institute, University of Chicago, Chicago, IL 60637, USA and ^5^Fellowship for the Interpretation of Genomes, Burr Ridge, IL 60527, USA

## Abstract

**Summary:** Annotation of metagenomes involves comparing the individual sequence reads with a database of known sequences and assigning a unique function to each read. This is a time-consuming task that is computationally intensive (though not computationally complex). Here we present a novel approach to annotate metagenomes using unique *k*-mer oligopeptide sequences from 7 to 12 amino acids long. We demonstrate that *k-*mer-based annotations are faster and approach the sensitivity and precision of blastx-based annotations without loosing accuracy. A last-common ancestor approach was also developed to describe the members of the community.

**Availability and implementation:** This open-source application was implemented in Perl and can be accessed via a user-friendly website at http://edwards.sdsu.edu/rtmg. In addition, code to access the annotation servers is available for download from http://www.theseed.org/. FIGfams and *k*-mers are available for download from ftp://ftp.theseed.org/FIGfams/.

**Contact:** redwards@mail.sdsu.edu

**Supplementary information:** Supplementary data are available at *Bioinformatics* online.

## 1 INTRODUCTION

Metagenomics has revolutionized microbial ecology. The extraction, purification and sequencing steps have been trivialized by next generation sequencing approaches, and environmental samples are routinely processed from collection to DNA sequence in a matter of days (Dinsdale *et al.*, 2008). The bottleneck in metagenomics approaches has become the analysis of the sequences. The computational comparison of sequences against all of the known proteins using blastx is limited by computational resources (Meyer *et al.*, 2008; Wilkening *et al.*, 2009).

Here, we describe a novel approach to analysing metagenomic sequences using unique signature *k*-mers that represent members of a protein family. Limited additional computational resources are required when the size of the underlying database doubles, as in the best case the search is dependent on the length of the *k*-mer. We implemented an API and Web servers to support the annotation of metagenomes using *k-*mers.

## 2 METHODS

Fellowship for the Interpretation of Genomes (FIG) protein families, FIGfams, were constructed as described previously (Meyer *et al.*, 2009). To identify the signature *k*-mers that represent members of a protein family, all amino acid oligomers from 7 to 12 amino acids were identified that were (i) present in one of more members of the FIGfam and (ii) were not present in any other FIGfam. These oligos are unambiguous representatives of the family. A binary tree was built to allow rapid searching of the *k*-mers and to identify their cognate protein families.

DNA sequences from metagenomes are assigned functions based on the FIGfams that match. To search the *k-*mers, the DNA sequence is translated in all six frames, and an exact matching algorithm is used to find identical amino acid strings. Requiring either multiple independent *k*-mer matches from a single family or a minimum number of *k*-mer matches over a minimum sequence length is used to adjust the sensitivity of the match. The search reports the first and last positions in the query sequence where the *k-*mers match, and the number of *k-*mers that match that region. These matches can be combined into subsystems (Overbeek *et al.*, 2005). The last common ancestor of the organisms in each family is also identified.

To validate whether the *k*-mer approach could be used to annotate metagenomes, simulated metagenomes were made from 70 different microbial genomes representing a diverse selection of organisms that had been annotated using Rapid Annotation Using Subsystems Technology (RAST) but had not been included in the FIGfams (Supplementary Table 1). The metagenomes were constructed with Grinder (Angly *et al.*, 2012) and were designed with median DNA fragment lengths of 30, 50, 75, 100, 250 and 500 bp. Metagenomes were annotated by searching for genes using the *k*-mers, and blastx searches of either the seed-nr database or the database of proteins used to generate the *k*-mer library. Not every protein in the seed-nr is included in a FIGfam (e.g. singleton proteins are not members of a family).

## 3 RESULTS

The most recent build of the FIGfams (Release 59 constructed July, 2012) contains 11 856 938 proteins organized into 178 208 families. The FIGfams have a mean of 66.5 protein members and a median of 6 protein members. Both the FIGfams and *k*-mers are available for download from ftp://ftp.theseed.org/FIGfams/. The coverage of oligomers is shown in Table 1.

**Table 1.** Statistics of the *k*-mers and FIGfams

*k*-mer size	Number of *k*-mers	Number of families	Mean number of *k-*mers per family	Median number of *k-*mers per family
7	207 362 319	171 606	1208.4	110
8	639 234 488	173 332	3687.9	325
9	812 679 565	173 513	4683.7	404
10	866 382 763	173 561	4991.8	425
11	896 943 566	173 587	5167.1	434
12	921 081 710	173 606	5305.6	441

The sensitivity and specificity of the *k*-mer approach was measured using synthetic metagenomes constructed using genomes that were not included in the FIGfams build (Supplementary Fig. 1). The sensitivity [TP/(TP + FN)] measures whether genes that are there (i.e. in the complete genome annotation) are found; for very short DNA sequences, most genes that are there are missed.

The *k*-mers are much more sensitive than blastx, finding almost 1/5 of genes present when the fragment lengths are only 30 bp. When the fragment length exceeds 50 bp, blastx performance improves rapidly as the high scoring pairs exceed the threshold for inclusion, and approaches that of the most sensitive *k*-mer searches. All approaches reach a sensitivity plateau once the sequence length exceeds 100 bp, and the best methods only find ∼70% of the genes on the fragments. For the longest fragment lengths (500 bp), the mean open reading frame length on each fragment was only 340 bp as genes may start and end off the fragment. In all cases, longer sequence reads resulted in more sensitive assignment of annotations to the DNA sequences, as seen before (Wommack *et al.*, 2008).

The precision [TP/(TP + FP)] reports whether too many genes are being called on a fragment. With very short fragments, both short *k*-mers and blastx overcall genes. However, longer *k*-mers result in more confident calls, regardless of fragment length. BLAST precision is improved by only using the set of confidently called proteins—those that are in families and used to make the *k*-mers. Accuracy measures whether the genes that are identified are correctly annotated (Overbeek *et al.*, 2005). This measure of accuracy is testing whether the function assigned by the best blastx hit or *k*-mer reflects of the ‘true’ function of the protein as annotated in the genome.

*k*-mer searches were on average 860 times faster than blastx because the *k*-mer approach neither extends the matches nor calculates alignment statistics for the resulting matches. DNA sequence annotations using *k-*mers are as sensitive, precise and accurate as blastx searches, especially for shorter reads. The main disadvantage of using unique *k-*mers is as the read-length increases the blastx sensitivity exceeds that of *k-*mers.

As shown in Supplementary Figure 1, the length of the *k-*mer has a strong impact on the precision, sensitivity and accuracy of the search. Based on these data and empirical observations, we recommend that users require at least two *k*-mer matchers per sequence (but generally no more than four *k*-mer matchers), and eight- or nine-amino acid *k-*mers. These parameters provide reasonable estimates of metagenome composition.

The *k-*mers represent a protein family, but not a specific organism from that family. However, most families only contain a few proteins (the median size of the protein families is only six), and thus most protein families only come from a handful of species and very few genera. To assign taxonomic groups to sequences, we identify the last common ancestor of the organisms whose proteins make up a family from their taxonomy. This is an approximation that provides for a rapid assessment of the members of the community.

The SEED annotation servers (http://servers.theseed.org/) provide programmatic access to the *k*-mer annotation algorithm via an API. These servers support the assignment of functions to protein or DNA sequences. Detailed examples are provided at that page and at http://edwards.sdsu.edu/RTMg. The Web interface was built to provide rapid interpretation of a metagenome sample. The samples are analysed in groups of sequences (currently the default is 10 000 sequences at a time), in a round-robin fashion. Users see the results of their annotation as it is being performed. At any time, all of the raw data can be downloaded as raw text to import into any other analysis platform or package. In practice we use this system to assess the quality of the metagenome and visualize similarities to the sample, leaving more detailed and thorough analysis until more time-consuming comparisons are complete. However, the results of the *k*-mer-based analysis are generally recapitulated in downstream analyses.
